# iVision MRSSD: A comprehensive multi-resolution SAR ship detection dataset for state of the art satellite based maritime surveillance applications

**DOI:** 10.1016/j.dib.2023.109505

**Published:** 2023-08-21

**Authors:** Muhammad Farhan Humayun, Farrukh Aziz Bhatti, Khurram Khurshid

**Affiliations:** iVision Lab, Department of Electrical Engineering, Institute of Space Technology, Islamabad, Pakistan

**Keywords:** Ship detection, Localization, Synthetic aperture radar (SAR), Multi resolution satellite images, YOLO Labels format, Ships dataset

## Abstract

This article describes a comprehensive Synthetic Aperture Radar (SAR) satellite based ships dataset for use in state of the art object detection algorithms. The dataset comprises 11,590 image tiles containing 27,885 ships examples. Each image tile has spatial dimensions of 512 × 512 pixels and is exported in JPEG format. The dataset contains a wide variety of inshore and offshore scenes under varying background settings and sea conditions to generate an all-inclusive understanding of the ship detection task in SAR satellite images. The dataset is generated using images from six different satellite sensors covering a wide range of electromagnetic spectrum including C, L and X band radar imaging frequencies. All the sensors have different resolutions and imaging modes. The dataset is randomly distributed into training, validation and test sets in the ratio of 70:20:10, respectively, for ease of comparison and bench-marking. The dataset was conceptualized, processed, labeled and verified at the Artificial Intelligence and Computer Vision (iVision) Lab at the Institute of Space Technology, Pakistan. To the best of our knowledge, this is the most diverse satellite based SAR ships dataset available in the public domain in terms of satellite sensors, radar imaging frequencies and background settings. The dataset can be used to train and optimize deep learning based object detection algorithms to develop generic models with high detection performance for any SAR sensor and background condition.

Specifications Table

**Table:** 

Subject	Computer Vision and Pattern Recognition, Remote Sensing
Specific subject area	Ship Detection in SAR Satellite Images, Satellite Remote Sensing
Type of data	Processed Images Corresponding text files for labels
How the data were acquired	Geo-referenced SAR Satellite images of six different space-borne sensors having high concentrations of ships were downloaded from European Space Agency (ESA) online portal, Capella space open dataset and ICEYE sample data and Sentinel Hub's EO browser under the creative commons (CC BY) license (http://creativecommons.org/licenses/by/4.0/).
Data format	Filtered
Description of data collection	The online resources mentioned above were scrutinized for the availability of high quality SAR satellite images with high concentrations of ships. A number of factors were taken into account for the selection of images including image spatial resolution, imaging mode and geographic location of the areas covered. Some of the areas covered include the Panama Canal, Suez Canal, Strait of Gibraltar, Port of Shanghai and Karachi Port. Moreover, online request forms briefly describing the title and purpose of the research were submitted to the European Space Agency (ESA) for accessing the satellite images of three out of six sensors.
Data source location	Source of primary data: Capella: https://registry.opendata.aws/capella_opendata ICEYE: https://www.iceye.com/downloads/datasets TerraSAR-X: https://tpm-ds.eo.esa.int/oads/access/collection/TerraSAR-X Paz: https://tpm-ds.eo.esa.int/oads/access/collection/PAZ Alos-PALSAR: https://alos-palsar-ds.eo.esa.int/oads/access/ Sentinel-1: https://apps.sentinel-hub.com/eo-browser/
Data accessibility	Repository name: Harvard Dataverse Repository Data identification number: https://doi.org/10.7910/DVN/0NUWST Direct URL to data: https://dataverse.harvard.edu/dataset.xhtml?persistentId=10.7910/DVN/0NUWST

## 1 Value of the Data

• The dataset is intended for the usage and exploration of possibilities for ship detection in multi-resolution SAR satellite images.
• This is a diverse dataset consisting of multi-spatial resolution images from six different SAR satellite sensors covering C, L and X bands of the radar imaging frequencies.
• The dataset showcases unique characteristics and challenges associated with SAR satellite images and can be used for training, validation and testing as well as modification and optimization of state of the art, deep learning based object detection models.
• The dataset contains 11,590 labeled tiles containing 27,885 ship examples. It includes a mix of inshore and offshore scenes under different sea conditions as well as complex backgrounds including land features such as ports, harbors, islands and sea clutter.
• Due to the diversity of the dataset, it can be used to develop generalized SAR ship detection models that can achieve high ship detection performance on any kind of SAR sensor under different background settings.

## 2 Objective

SAR satellite sensors are considered excellent for maritime applications as they can image during both day and night times. Moreover, they are also unaffected by cloud coverage and hence can provide unrestricted coverage over the coastal areas [1]. Apart from that they have wider swath coverage as compared to their optical counterparts which means that they can cover large areas within less amount of time [2,3]. Despite these benefits, there still remain various challenges associated with SAR images which make SAR based ship detection a relatively arduous task. These include various distortions and de-shaped object boundaries due to uneven scattering phenomenon, issues of inter-class similarity signature problems i.e. two very different objects can seem to be similar when seen in a SAR image, weak generalization ability of optical object detection models on SAR data and scarcity of publicly available satellite based SAR datasets as compared to the optical datasets. To address some of these issues, we propose a comprehensive multi-resolution satellite based SAR ship detection dataset which can be used to train, test and validate state of the art deep learning based object detection models.

## 3 Data Description

### 3.1 Comparison with publicly available SAR ship datasets

Currently, there are only five significant satellite based SAR ship datasets available in the open source. These include SSDD [4], SAR-Ship-Dataset [5], Air-SARShip-1.0 [6], HRSID [7] and LS-SSDDv1.0 [8]. Another dataset named SRSDD-v1.0 [9] contains rotated bounding boxes instead of regular upright bounding boxes. Table 1 shows a comparison among various datasets available in the open source along with the proposed dataset. Based on the comparisons in Table 1, it is evident that the proposed MRSSD dataset is most diverse in terms of the number of SAR satellite sensors used, spatial resolutions as well as the range of radar imaging frequency bands covered.

**Table 1:** Comparison of publicly available SAR Ship detection datasets with the proposed iVision-MRSSD dataset.

	SSDD	SAR Ships Dataset	Air-SAR Ship-1.0	HRSID	L-SSDD V1.0	SRSSD	MRSSD (Proposed)
Year of Publication	2017	2019	2019	2020	2020	2021	**2023**
No. of image tiles	1160	43,819	31	5604	9000	666	**11,590**
Tile size	Variable	256 × 256	3000 × 3000	800 × 800	800 × 800	1024 × 1024	**512** × **512**
No. of sensors used	03	02	01	03	01	01	**06**
Frequency bands covered	C, X	C	C	C, X	C	C	**C, L, X**

### 3.2 MRSSD description

**Fig. 1:**
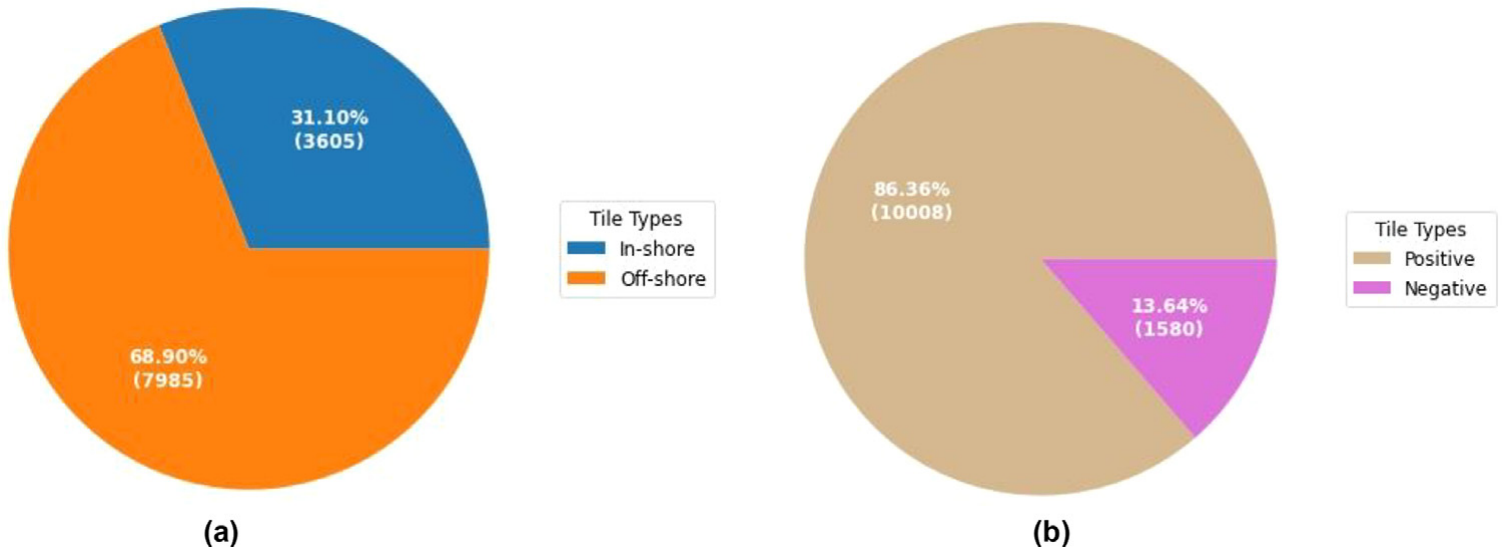
(a) Distribution of in-shore and off-shore tiles. (b) Distribution of positive and negative tiles.

The proposed Multi-Resolution SAR Ship Detection dataset contains 11,590 images along with the same number of label files. The image format is JPEG, whereas the label files are in text format. Each image tile has a standard height and width of 512 × 512 pixels. The dataset contains 27,885 ships of varying sizes ranging from small boats to large container ships. MRSSD has a ship/image ratio of 2.405 which means that on average, each image contains about 2.405 ships. Moreover, the dataset contains a mix of inshore and offshore images with varying background scenes including open sea, islands, ports, harbors and sea clutter. These different background settings can help the detection models in learning generic features and perform better under different conditions. An image that includes any form of land feature is termed as an inshore scene, whereas images that solely contain water in the background are called offshore images. Fig. 1(a) shows the distribution of inshore and offshore tiles in the dataset. There are 7985 offshore tiles which make up the bulk of the dataset. The number of inshore tiles is 3605. The dataset also contains certain negative examples. These include those images which do not contain any ship. These negative examples can help the object detection models in developing an understanding of the pure background scenes. Fig. 1(b) shows the distribution of positive and negative images in the dataset.

The dataset is based on images from six different SAR satellite sensors. The satellite sensors include Capella Space [10], ICEYE [11], TerraSAR-X [12], Paz [13], Alos-PALSAR [14] and Sentinel-1 [15]. Table 2 summarizes the detailed specifications of satellite sensors used for preparation of the proposed dataset.

**Table 2:** Specifications of satellite sensors used in iVision: MRSSD Dataset.

Satellite Name	Resolutions/Mode of Operations	Imaging Frequency	Polarization Modes	No. of Images used	Sensor Operating Period	Sensor Operator and Country
Capella	Spotlight: 0.5 m S-Spotlight: 0.8m Strip map: 1.2 m	X-Band: 9.4 - 9.9 GHz	Single	11	2020 - Present	Capella Space - USA
ICEYE	Spot: 1 m Strip: 3 m Scan: 15 m	X-Band: 9.65 GHz	Single	08	2019 - Present	ICEYE - Finland
TerraSar-X	Spotlight: 1 m Strip map: 3 m ScanSAR: 18.5 m Wide Scan: 40 m	X-Band: 9.65 GHz	Single, Dual, Quad	08	2007 - Present	German Aerospace Center (DLR) - Germany
Paz	Spotlight: 1 m Strip map: 3 m ScanSAR: 18.5 m Wide Scan: 40 m	X-Band: 9.65 GHz	Single, Dual	08	2018 - Present	Hidesat - Spain
Alos-PALSAR	Fine Beam: 10 m ScanSAR: 100 m	L-Band: 1.27 GHz	Single, Dual, Quad	14	2006 - 2011	JAXA - Japan
Sentinel-1	Strip map: 5 m Wide Swath:10 m Extra Wide: 40 m	C-Band: 5.405 GHz	Single, Dual	10	2014 - Present	Thales Alenia Space - Italy/ESA

**Fig. 2:**
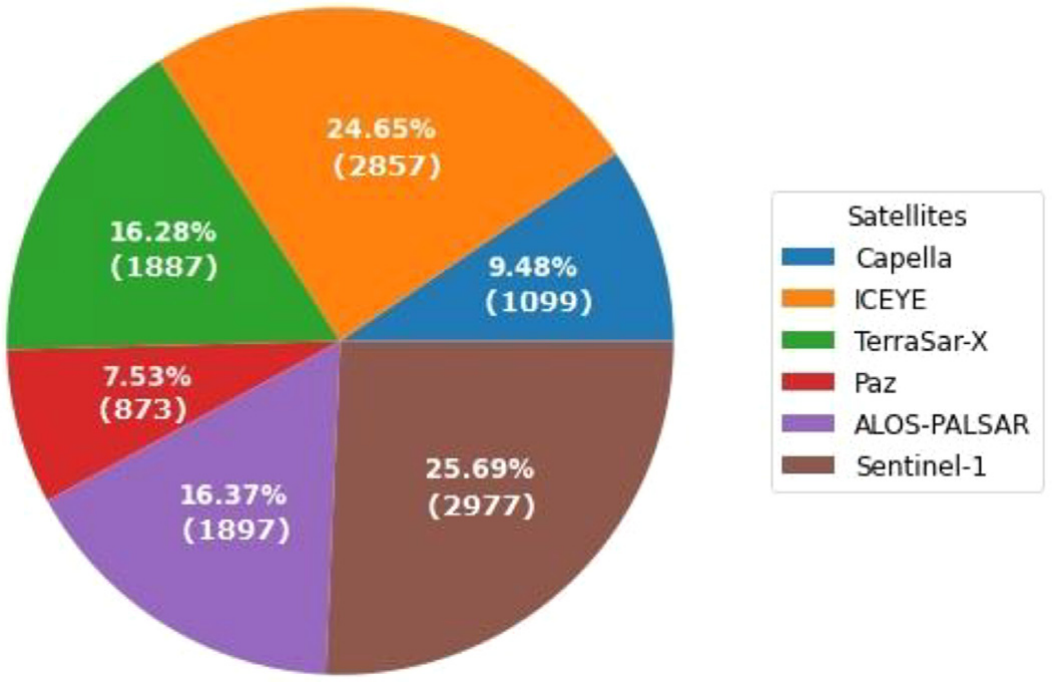
Distribution of the image tiles in terms of various satellite sensors involved.

**Fig. 3:**
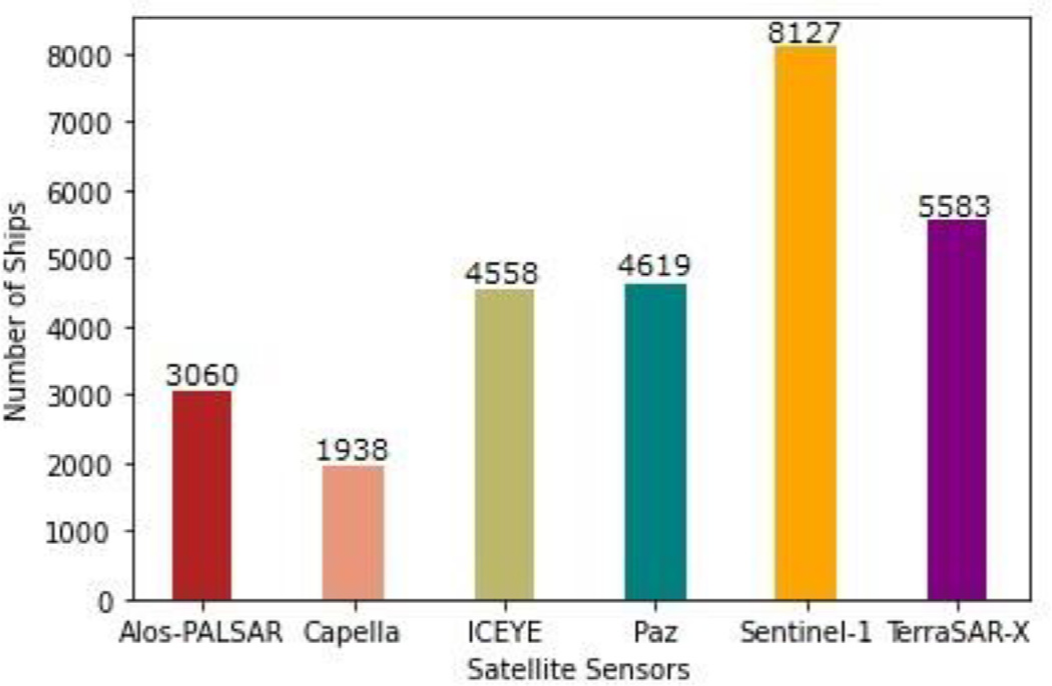
Distribution of Ships in terms of various satellite sensors involved.

Fig. 2, Fig. 3 show the distribution of image tiles and number of ships, respectively, in terms of satellite sensors involved. It can be seen that the European satellite, Sentinel-1 has the most number of tiles, whereas the Spanish satellite, Paz has the least number of tiles in the dataset. Similarly, the most number of positive ship examples belong to Sentinel-1 as well, whereas least number of ship examples are from Capella.

**Fig. 4:**
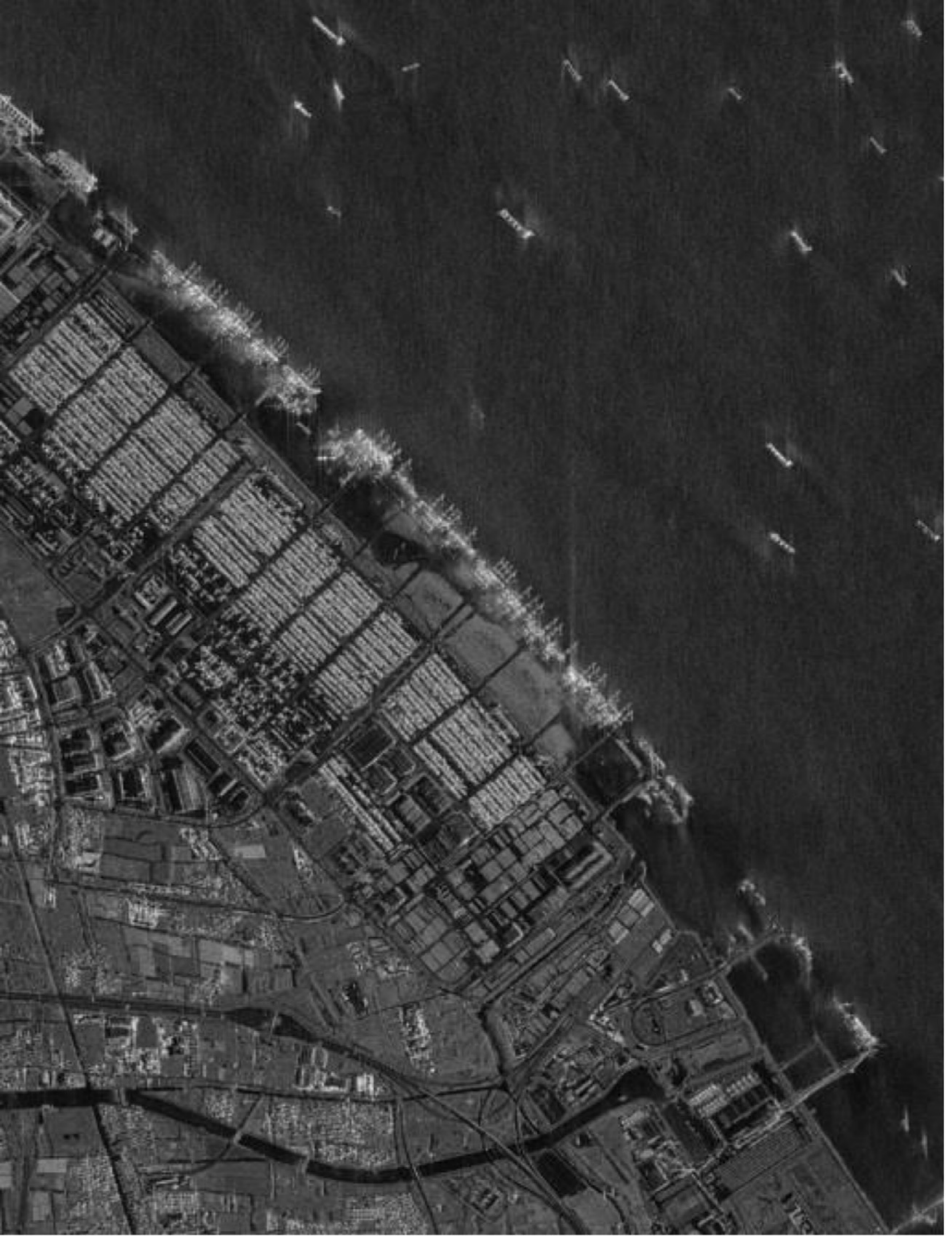
Sample large scale image of the Bay of Gibraltar captured by Capella satellite.

Fig. 4 shows a sample large scale image of the Bay of Gibraltar captured by Capella satellite.

**Fig. 5:**
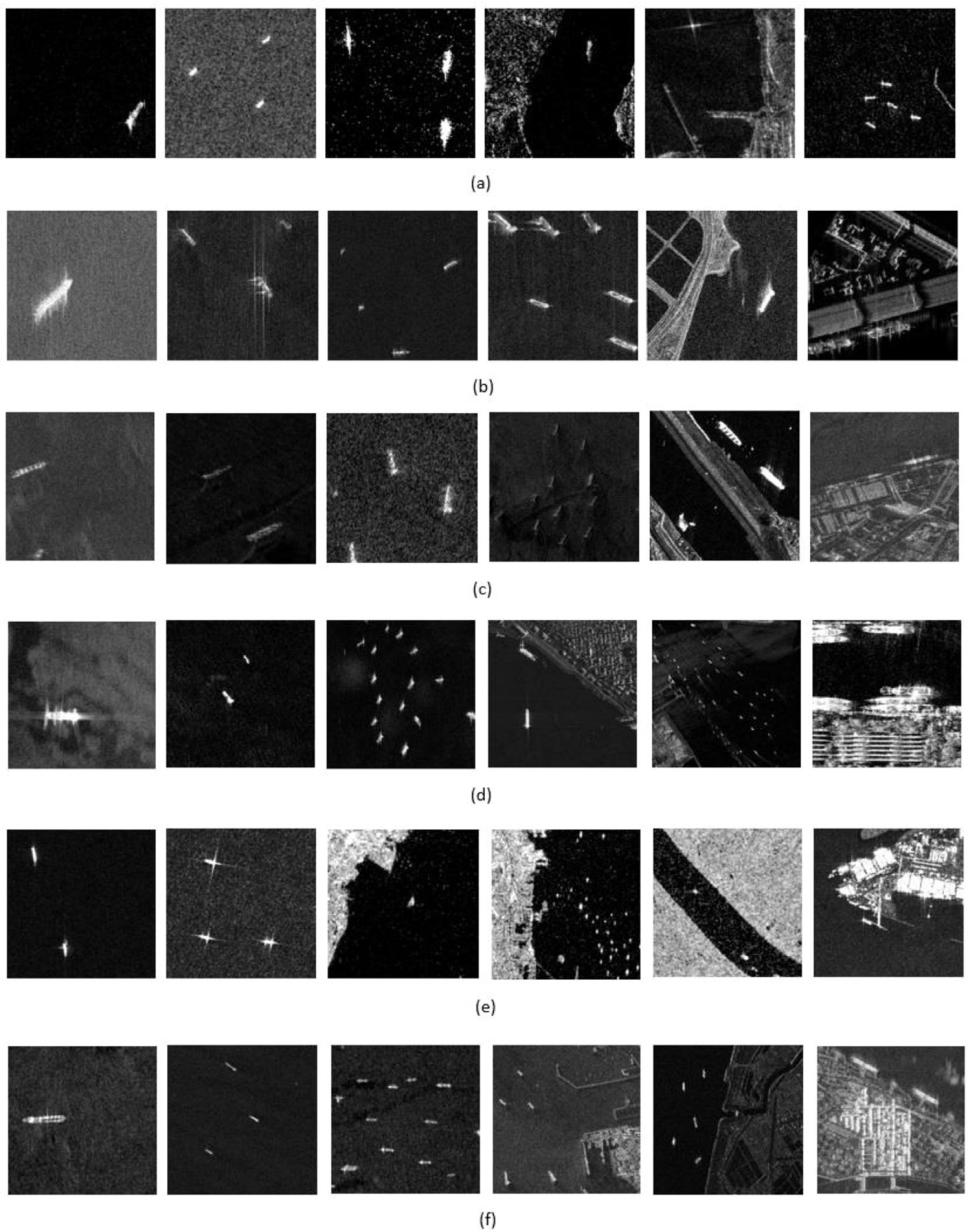
(a–f) Sample image tiles of ALOS-PALSAR, Capella, ICEYE, Paz, Sentinel-1 and TerraSar-X respectively.

Fig. 5 shows the sample 512 × 512 tiles of all the satellite sensors used in the dataset. It can be seen that the dataset contains a mix of inshore (complex) and offshore (relatively simple) scenes. Moreover, it is also evident that the ships have varying sizes and dimensions as well.

**Fig. 6:**
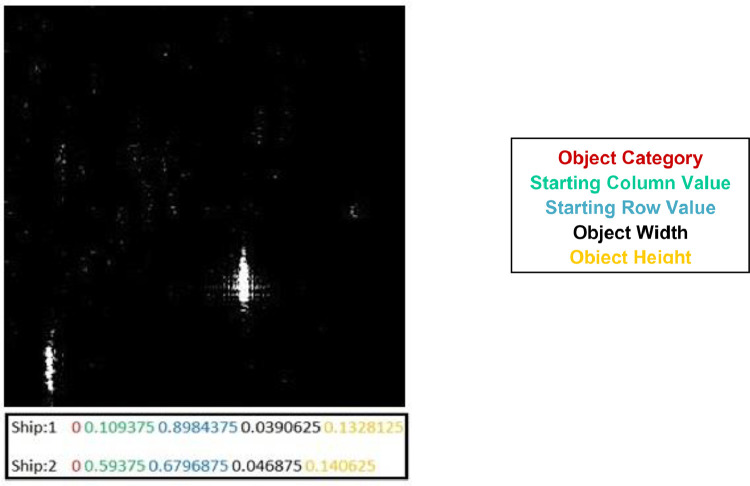
Sample image tile along with corresponding ship labels.

The dataset labels follow the YOLO5 series annotation format where a text file is generated against each image tile with the same name. The text files consist of five different fields containing information regarding object category, normalized row and column values of the object as well as its normalized height and width. Fig. 6 depicts a sample data tile along with its labels.

**Fig. 7:**
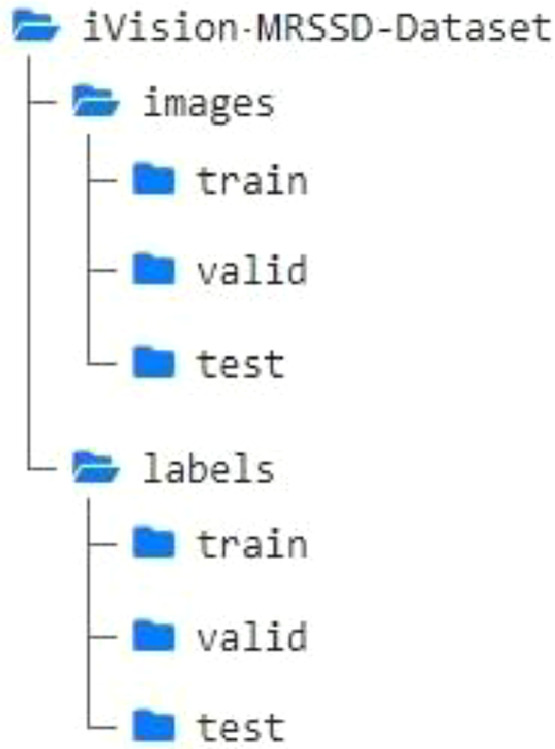
Directory structure of the iVision-MRSSD Dataset.

The dataset is randomly split into training, validation and test sets in the ratio of 70:20:10, respectively. This is done to achieve standardized sets for the purpose of comparison and benchmarking on various object detection models. Fig. 7 shows the structure of the data folder depicting the root directory and its sub-folders.

## 4 Experimental Design, Materials and Methods

### 4.1 Acquisition of satellite images

All the satellite images used were acquired from online resources under the creative commons (CC BY) license. Images of Capella Space, ICEYE and Sentinel-1 were accessed and downloaded from direct open access archives of respective satellites. Whereas for images pertaining to TerraSAR-X, Paz and Alos-PALSAR, online request forms describing the purpose for the use of satellite images were submitted to European Space Agency (ESA) for access to their online data archives. The respective data archives were searched for the availability of desired satellite images with high concentration of ships. Consequently, selected images were downloaded in TIFF and GRD formats.

### 4.2 Data pre-processing and generation of training tiles

These images were processed in specialized GIS software including ArcGIS and SNAP. Specifically, histogram equalization, brightness/contrast adjustment and noise reduction techniques were applied for enhancement of ships against image backgrounds and suppression of unwanted noise. The processed images were then exported in high resolution JPEG format. Further, all the images were sliced into 512 × 512 tiles using Python script to achieve standard dimensions capable of being handled by deep learning based object detection models. Tiles pertaining to edge and corner cases where the dimensions were not exactly 512 × 512 pixels were removed. Moreover, the images were sliced such that each tile has a 50 percent overlap with the subsequent tile horizontally as well as vertically.

### 4.3 Dataset labeling and verification

After the generation of trainable tiles, all the tiles were carefully labeled with the help of SAR imagery and maritime domain experts. An open source tool named Modified Open Labeling [16] was used to label the whole dataset. Moreover, each individual labeled tile was verified for correctness of labels using the open source tool named draw YOLO box [17].

### 4.4 Splitting the dataset

The dataset was randomly divided into train, validation and test sets in the ratio of 70: 20: 10, respectively, using Python based library split-folders [18].

## Ethics Statements

All the satellite images were acquired with the consent of relevant data owners under the creative commons (CC BY) license (http://creativecommons.org/licenses/by/4.0/).

Expert guidance was received from SAR and maritime domain experts with their consent and were informed in advance about the purpose and objectives of the research.

## CRediT authorship contribution statement

**Muhammad Farhan Humayun:** Conceptualization, Methodology, Software, Formal analysis, Investigation, Data curation, Writing – original draft, Visualization. **Farrukh Aziz Bhatti:** Methodology, Investigation, Resources, Writing – review & editing, Project administration, Funding acquisition. **Khurram Khurshid:** Validation, Resources, Writing – review & editing, Project administration, Supervision, Funding acquisition.

## Declaration of Competing Interest

The authors declare that they have no known competing financial interests or personal relationships that could have appeared to influence the work reported in this paper.

## Data Availability

iVision-MRSSD: Multi-Resolution Satellite based SAR Ship Detection Dataset (Original data) (Dataverse).
